# Parafoveal Microperimetric Retinal Sensitivity as a Key Parameter Associated with Vision Loss in Retinitis Pigmentosa

**DOI:** 10.3390/diagnostics14232691

**Published:** 2024-11-29

**Authors:** Yu-Ting Hsiao, Hsiu-Mei Huang, Ta-Ching Chen, Jung Lo, Yung-Jen Chen, Hsi-Kung Kuo, Jong-Jer Lee

**Affiliations:** 1 Department of Ophthalmology, Kaohsiung Chang Gung Memorial Hospital and Chang Gung University College of Medicine, Kaohsiung 83340, Taiwan; yuting1008@cgmh.org.tw (Y.-T.H.); sammy1013@cgmh.org.tw (H.-M.H.); enchantvik@gmail.com (J.L.); f75622@cgmh.org.tw (Y.-J.C.); hsikung@cgmh.org.tw (H.-K.K.); 2 Department of Ophthalmology, National Taiwan University Hospital, Taipei 10041, Taiwan; tachingchen1@ntu.edu.tw; 3 Center of Frontier Medicine, National Taiwan University Hospital, Taipei 10041, Taiwan; 4 Center for Mitochondrial Research and Medicine, Kaohsiung Chang Gung Memorial Hospital, Kaohsiung 83340, Taiwan

**Keywords:** retinitis pigmentosa, microperimetry, visual function, fundus autofluorescence

## Abstract

**Background:** Although optical coherence tomography (OCT) is useful in determining outer retinal architecture, it may be suboptimal when monitoring subtle changes in retinitis pigmentosa (RP) patients. The aim of this study is to investigate precise microperimetric parameters for disease severity identification in RP patients. **Methods:** A cross-sectional and retrospective study. Thirty-nine eyes of 39 RP patients were included. Associations between logMAR visual acuity (VA), spectral-domain OCT, fundus autofluorescence imaging (FAF), and various microperimetric measures were evaluated. Microperimetric test locations were grouped into “foveal”, parafoveal “inner ring”, and perifoveal “outer ring”. Independent variables were analyzed based on logistic regression, then assessed using area under the receiver operating characteristic curve (AUROC). **Results:** Among all microperimetric measures, linear regression analysis indicated that mean retinal sensitivity and deep scotoma count at the parafoveal inner ring were the principal parameters associated with decreased VA. The AUROC was highest for deep scotoma count at the inner ring at a value of 0.829, with the cut-off point at 3.5. A visual function index was then established according to the number of parafoveal deep scotoma points, in order of mild (0 points), moderate (1–3 points), and severe (4 or more points). Our microperimetric visual function index also correlated significantly to logMAR VA and previously established FAF patterns. **Conclusions:** Our study discovered deep scotoma count at the parafoveal inner ring to be a key microperimetric parameter in evaluating vision loss in RP patients. Those with four or more deep scotoma points at the parafoveal inner ring are more likely to have functional low vision.

## 1. Introduction

Retinitis pigmentosa (RP) is the most common inherited retinal disease (IRD) typically presenting with reduced night vision and progressive peripheral visual field loss [1]. It is characterized by the gradual loss of photoreceptor integrity in the outer retina, beginning with the loss of rod photoreceptors, then advancing to cone photoreceptor involvement in the late stages [2]. Due to the diversity of RP, genetic testing has also become an important strategy to complement clinical findings and clarify diagnosis [3]. As a result, morphological and functional evaluations of retinal changes can be beneficial in assessing the disease course and remaining retinal function in RP patients.

Optical coherence tomography (OCT) is a typical method in retinal disease analysis, and several studies have reported the use of OCT to follow ellipsoid zone (EZ) changes in RP patients [4,5,6]. However, there is difficulty in evaluating transverse structural OCT changes and their correlation to visual acuity (VA), especially in advanced stages once EZ integrity is lost [4]. Meanwhile, fundus autofluorescence (FAF) imaging provides information not evident through conventional fundus photos by enabling lipofuscin visualization at the level of the retinal pigment epithelium (RPE) cell monolayer [7,8]. FAF is a convenient, fast, and noninvasive imaging procedure, and its potential as a prognostic marker has been shown to identify distinctive distributions in retinal dystrophies [8,9].

IRD patients have variable losses of best-corrected visual acuity (BCVA), which can range from 20/20 to 20/400 or worse [9]. In patients with RP, the central vision is usually preserved, meaning their VA remains unaffected, in early-to-moderate disease stages. Vision begins to deteriorate once the retinal degeneration encroaches centrally, resulting in severe sight impairment [1,10]. In addition to changes in the retina, the optic nerve head (ONH) may become waxy and pallor in advanced stages, but its relationship to VA remains to be determined [11]. Although a significant difference was observed in the VA of patients with and without pale ONH appearance, no significant correlation was seen in the VA of RP patients with or without retinal nerve fiber layer (RNFL) thinning [11,12]. Functionally, VA is the gold standard reference method for assessing visual performance. However, VA may not reflect extra-foveal focal macular dysfunction and the functional impact of RP on the patient’s eyes and quality of life [13]. Therefore, exploring additional measures beyond VA may help detect subtle visual function changes in those with visual loss. 

Many standard clinical visual function measurements are currently available, including visual field assessments and visual electrophysiology [14]. While visual field testing is better at identifying patterns of visual loss, it is time consuming, requires specially trained personnel, and is often complicated by unstable fixation in retinal dystrophy patients [15]. Though full-field electroretinography provides an overall retinal function evaluation, it is often insensitive to subtle changes in retinal sensitivity [16]. The identification of appropriate tools that can comprehensively assess visual function in RP patients is of paramount importance.

Microperimetry has been utilized to test visual function in various RP research studies [17,18,19]. Microperimetry-3 (MP-3, Nidek Co., Aichi, Japan) is a fundus-controlled perimetry, and provides a spatial assessment of retinal function across the macula by presenting visual sensitivity test points on a fundus image via eye tracking [20,21,22]. It has greater precision and resolution than standard perimetry and can also be correlated with structural evaluation modalities [23]. By reducing the bias of the MP-3 examination through an improved motion tracking system and a fully automatic measurement procedure, no clinically relevant learning effect of the MP-3 was found in test–retest reproducibility, even in patients with impaired retinal function [24]. On the contrary, significant learning effects have been shown for conventional perimetry and MP-1 microperimetry, the former version of MP-3 [25,26]. Research on IRD has proven MP-3 as a reliable approach for analyzing macular structure–function correlation, along with OCT [21,27,28,29]. 

The MP-3 offers a broad range of examination parameters. However, there are no studies on which microperimetric parameter has the most prognostic value in RP patients to allow for practical use of this tool in clinical practice. In this study, our main goal is to explore the functional association between VA and different retinal functional parameters evaluated with MP-3, supported by anatomical changes on OCT and FAF. Our aim is to establish a more precise and straightforward microperimetric variable for functional evaluation in RP patients.

## 2. Materials and Methods

### 2.1. Patients and Study Design

This cross-sectional study was conducted at Kaohsiung Chang Gung Memorial Hospital between 1 October 2022 and 30 September 2023. The study was approved by the Committee of Medical Ethics and Human Experiments of CGMH, Taiwan (IRB approval no. 202200772A3), and all research procedures have been carried out in accordance with the Declaration of Helsinki and the ARVO statement for experiments involving humans. All individuals gave their informed consent.

Diagnosis of RP was based on nyctalopia, pigmentary retinal changes, visual field constriction, full-field scotopic electroretinography, and whole exome sequencing (WES), where available. Exclusion criteria were patients with media opacities that interfered with image acquisition, high myopia (>6 diopters), glaucomatous optic neuropathy, and presence of macular edema related to neovascular age-related macular degeneration, diabetic macular edema or retinal vein occlusion. Ophthalmological examination included assessment of Snellen VA (logMAR VA), fundus photography, spectral-domain OCT (SD-OCT), FAF, and MP-3. The right eye of each patient was selected for analysis, and classified into two groups, the fair VA and poor VA group. We categorized subjects into the poor VA group based on the World Health Organization’s (WHO) criterion of moderate visual impairment or worse, often known as low vision. This was defined as individuals presenting with a VA of LogMAR 0.5 and above [30]. 

### 2.2. Fundus Autofluorescence and Spectral-Domain Optical Coherence Tomography

FAF and fundus photography was obtained using an Optos (Optos Inc., Malborough, MA, USA). FAF images were classified into large ring (≥3° radius), small ring (<3° radius), patch, and central hypoautofluorescence (atrophy), and measurements of structures on FAF were carried out as previously established [31]. Macular SD-OCT was obtained with Heidelberg Spectralis^®^ HRA + OCT (Heidelberg Engineering, Heidelberg, Germany) to determine the central macular thickness (CMT) and the presence or absence of EZ disruption involving the fovea. The CMT was assessed by the imaging protocol provided by the device. Two trained retinal specialists (Y.T.H. and Y.J.C.) reviewed the FAF and OCT scans independently masked to other clinical data, and these were evaluated by a third retinal specialist (J.J.L.) in case of disagreement.

### 2.3. Fundus-Controlled Microperimetry

**Figure 1:**
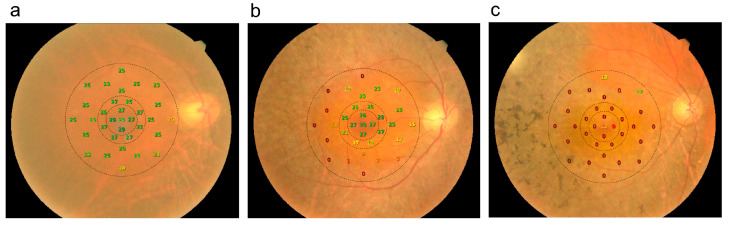
Schematic representation of 3 zones including the fovea, parafoveal inner ring, and parafoveal outer ring (dotted lines) on microperimetry. (**a**) The right eye of a 55-year-old female with mean sensitivity of 23.0 dB and best-corrected visual acuity (BCVA) of 20/30. (**b**) The right eye of a 65-year-old male with BCVA of 20/40 and mean retinal sensitivity of 15.5 dB. (**c**) The right eye of a 72-year-old woman with BCVA of 20/70 and mean retinal sensitivity of 1.6 dB.

The MP-3 microperimeter was used to perform testing after pupil dilation with topical 1% tropicamide and 0.5% mydriacyl. Sensitivity was tested in 33 macular test locations in the central 10° of retina using stimuli of the size of the Goldmann III target with a length of 200 milliseconds and a 4-2 strategy on a white monochromatic background. All tests were carried out in nearly dark (mesopic) light conditions. Each test was performed monocularly, with the contralateral eye patched. All participants underwent a training examination before the official microperimetry testing. Test reliability was assessed based on a metric consisting of the sum of false-positives and false-negatives divided by the sum of all catch study presentations; tests with a threshold of >20% were discarded and repeated, where possible [32,33]. The central 5 points were within 1.7° of the anatomical fovea and were referred to as “foveal”; the 8 perifoveal points were 3.5° to 4.7° from the center of the anatomical fovea and were referred to as the “inner ring”; and the remaining testing locations 5.6° to 10.1° from the fovea were referred to as the “outer ring.” The schematic representation of the three zones mentioned above are shown in Figure 1.

The retinal sensitivity at each location was determined by altering the light intensity iteratively until the dimmest visible stimulus was discovered. The sensitivity for each test location was established on a range of 0 dB to 34 dB. Test locations with 0 dB, meaning that only the brightest stimulus was recognized or no stimulus was registered at all, were classified as “deep scotoma”, whereas those with more than 0 dB but less than 12 dB were classified as “relative scotoma”. “Normal” test locations are those with sensitivity levels of 12 dB or above [21]. Using the NAVIS-EX software version 1.11.1.1, the average retinal sensitivity of the 33 stimuli (10° of retina) was determined.

### 2.4. Statistical Analyses

In descriptive analyses, categorical data were expressed as numbers and percentages, while quantitative variables were displayed as means ± standard deviations. The independent sample *t*-test was utilized to compare continuous variables, while the Fisher’s exact or Pearson’s chi-squared (χ^2^) tests were used to assess categorical data, as appropriate. Odds ratios (ORs) and 95% confidence intervals (CIs) were calculated using logistic regression analyses in which the VA group was the dependent variable. Significant predictors from the logistic regression analysis were considered independent variables in the multiple logistic regression analysis using a stepwise forward selection method. Receiver-operating characteristic (ROC) curves were generated by categorizing RP patients into two groups of fair and poor VA. The areas under the ROC curves (AUCs) were determined and compared with the AUC under the reference line. To establish the cut-off points of the scores, an ROC curve analyses estimated with the Youden Index was utilized. The result was deemed significant when the *p* value was less than 0.05. Statistical analyses were performed in SPSS version 22.0 for Windows (IBM, Armonk, NY, USA) and GraphPad Prism version 10.1.2 (GraphPad software Inc., San Diego, CA, USA).

## 3. Results

### 3.1. Patient Characteristics

**Figure 2:**
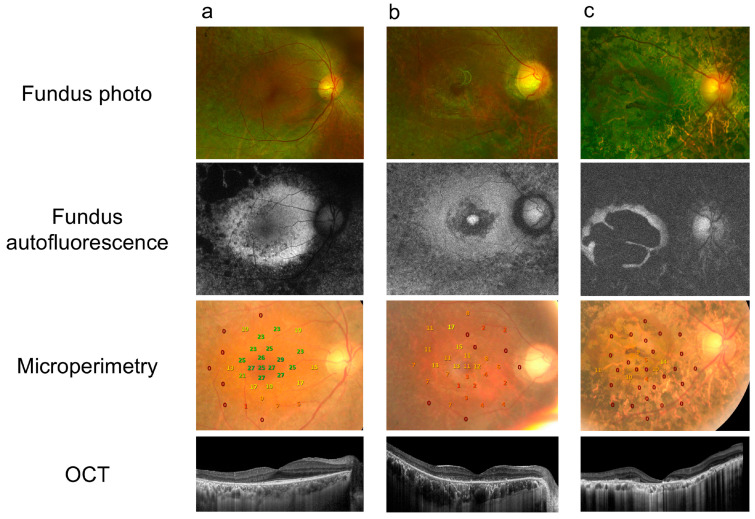
Representative cases of different fundus autofluorescence patterns in RP patients in this study. Fundus photographs, optical coherence tomography (OCT), and autofluorescence combined with microperimetry are shown. (**a**) Large ring: a 65-year-old male presenting with VA of 20/40 in the right eye. (**b**) Small ring: a 54-year-old female with VA of 20/100 in the right eye. (**c**) Patchy atrophy: a 45-year-old male presenting with VA of 20/200 in his right eye.

A total of 39 eyes from 39 RP patients were included in this study. Twenty-one subjects underwent WES, and *EYS* (24%) was identified as the most frequent disease-causing variant (Figure S1). The mean age was 48.03 *±* 15.52 years, and the mean logMAR VA was 0.79 *±* 0.70. The mean logMAR VA among patients in the fair VA group was 0.28 *±* 0.04, and 1.38 *±* 0.15 in the poor VA group. No differences in age or gender between those with fair vs. poor VA were found (Table 1). Representative cases of RP patients as imaged by fundus photographs, FAF, MP-3, and OCT are displayed in Figure 2.

### 3.2. Correlations of Structural Evaluations on OCT and FAF with VA

In eyes with poor VA, OCT revealed significantly thinner CMT and higher proportion of eyes with EZ disruption involving the fovea (Table 1), which is consistent with previous studies [4,5], further verifying our categorization method using the WHO definition [30]. Choroidal thickness was not significantly different between the two groups. The three main FAF patterns [31] were recognized with significant differences in distribution between the two VA groups. A large hyperautofluorescent ring was seen in 11/21 (52.4%) in the fair VA group, while foveal hypoautofluorescence patches and atrophy were seen in 9/18 (50%) in the group with poor VA (Table 1).

### 3.3. Measures of Macular Sensitivity and Their Association with VA

Table 1 and Table 2 summarize the association of overall microperimetric testing between the two VA groups. The poor VA group had significantly lower mean retinal sensitivity (*p* = 0.001) and worse fixation stability at circular regions within 2° and 4° diameters (*p* = 0.002 and *p* = 0.005, respectively) (Table 1). The number of deep scotoma points and normal test locations was significantly different between the fair VA and poor VA group. A higher number of deep scotoma points in the fovea, inner ring, and outer ring was found in the poor VA group, while the number of normal test points was lower in all three locations (Table 2). 

The associations of microperimetric parameters with logMAR VA in both univariate and multivariate linear regression are shown in Table 3. In the multivariate analysis integrating microperimetric parameters, lower mean retinal sensitivity (*β* = −0.406; *p* = 0.01) and a higher number of deep scotoma points (*β* = 0.354; *p* = 0.023) were associated with decreased logMAR VA (Table 3).

**Figure 3:**
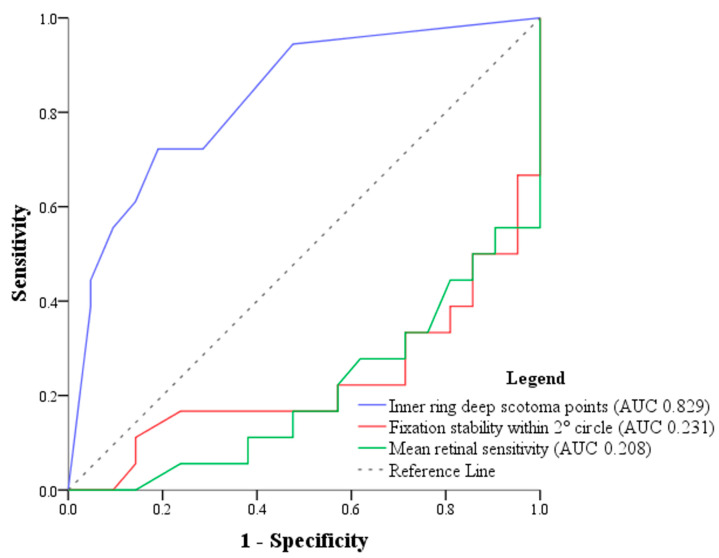
Receiver operating characteristic (ROC) curves and area under the curve (AUC) of significant microperimetric variables. Blue line: number of inner ring deep scotoma points (AUC = 0.829), the cut-off point for number of deep scotoma points at the inner ring was 3.5; red line: fixation stability within the 2° diameter circle (AUC = 0.231); green line: mean retinal sensitivity (AUC = 0.208). The dotted line represents the reference line (no discrimination).

On multivariate logistic regression analysis accounting for significant variables, the number of deep scotoma points at the inner ring was the only feature that was significantly associated with fair or poor VA based on a stepwise forward selection method (standard error: 0.141, odds ratio [95% confidence interval]: 1.561 [1.185–2.057], FDR-corrected *p* value = 0.002). The ROC curves of representative microperimetric variables, including the number of inner ring deep scotoma points, fixation stability within 2° diameter circle, and mean retinal sensitivity, were drawn. The AUCs were 0.829, 0.231, and 0.208, respectively (Figure 3). The cut-off point for number of deep scotoma points at the inner ring was 3.5. 

### 3.4. Establishing a Microperimetric Visual Function Index

**Figure 4:**
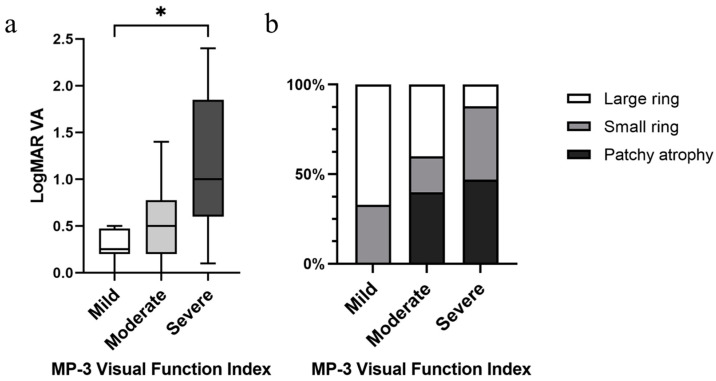
Significant correlations between MP-3 severity grading with (**a**) logMAR VA (*p* = 0.01) and (**b**) FAF patterns (*p* = 0.016). MP-3 severity grading is classified as mild (zero deep scotoma points at the inner ring, *n* = 12), moderate (less than 4 deep scotoma points at the inner ring, *n* = 10), and severe (4 or more deep scotoma points at the inner ring, *n* = 17). * *p* < 0.05.

We established a microperimetric functional index according to the number of deep scotoma points at the parafoveal inner ring. Patients with no deep scotoma points at the inner ring were considered relatively normal at the parafoveal area, and thus classified as mild. The moderate (1–3 deep scotoma points at the inner ring) and severe (4 or more deep scotoma points at the inner ring) classifications were defined based on the cut-off point from our ROC curve. Our microperimetric visual function impairment index also significantly correlated to logMAR VA and previously established FAF patterns (Figure 4a,b).

## 4. Discussion

Since there are many standard visual function measurements currently available and all with broad ranges of examination parameters, it is important to identify practical measures that are clinically meaningful and can reflect disease status efficiently and in a straightforward manner. To the best of our knowledge, this study is the first to evaluate and compare the various functional factors of microperimetry altogether in RP patients. Our work has established a specific microperimetry variable—the number of deep scotoma points at the parafoveal inner ring—to be a key visual loss parameter in patients with RP.

MP-3 enables a more comprehensive evaluation of visual performance not only in the fovea but across the macula, making MP-3 able to reflect daily living activities and quality of life meaningfully [34,35,36]. Microperimetry has also been established as a potential outcome measure for gene therapy clinical trials [21,27]. The eye tracker ensures that macular pathology and functional impairments in the MP-3 are precisely correlated [21]. A previous study reported retinal sensitivity to better reflect the magnitude of structural damage observed with OCT in RP [28], while another study mentioned deep scotoma counts to be more significantly associated to autofluorescence signal changes when compared to mean retinal sensitivity [37]. Thus, the precise selection of microperimetric indices is essential. In our results, only mean retinal sensitivity and the number of deep scotoma points at the inner ring demonstrated significant associations in both univariate and multivariate analyses (Table 3). When compared to earlier investigations [3,4], structural correlations with OCT and FAF revealed consistent trends among those with visual loss, confirming the feasibility of our VA grouping strategy using the WHO criterion [30]. Upon comparing RP patients with fair and poor VA, our analysis showed that only the number of deep scotoma points in the inner parafoveal ring remained significant after regression analysis. This is compatible with a previous cohort study that reported counting the number of deep scotoma points as an indicator of functional impairment extent [37]. Our study further indicates the specificity of the macular region—the parafoveal inner ring—in determining whether the patient has functional low vision.

In our study, ROC curves were analyzed to detect the specificity and sensitivity of various commonly used microperimetric parameters. When assessed individually, the number of deep scotoma points at the parafoveal inner ring had the largest area under curve (AUC = 0.829) compared to fixation stability at 2 degrees and mean retinal sensitivity (AUC = 0.231 and 0.208, respectively) (Figure 3). We found the cutoff value of the number of deep scotoma points at the inner ring to be 3.5, and with a sensitivity and specificity of 72 and 81%, respectively. Therefore, RP patients with four or more deep scotoma points at the inner ring on microperimetry is an indicator of having poor VA. In other words, we postulate that once the patient’s MP-3 testing starts nearing four deep scotoma points at the inner ring, we should be cautious of an impending symptomatic decline of visual performance, as this may be a critical minimum before central visual loss happens. Further longitudinal studies may be needed to confirm this finding. 

Subsequently, we set up a microperimetric functional index based on the number of deep scotoma points at the parafoveal inner ring. An advantage of deep scotoma count is that it can be translated into an area of autofluorescence, and the area covered by each point is around 0.42 mm^2^. Therefore, besides corresponding our microperimetric visual function index to logMAR VA, we also correlated topographic changes in FAF to our visual function index, to provide better structure–function verifications. Our microperimetric visual function index showed a significant increase in logMAR VA from the mild to severe groups, with also a significant rise in distribution from large to small FAF rings to patchy atrophy patterns (Figure 4a,b). A hyperautofluorescence ring indicates an early stage of disease with fairly preserved central vision and photopic function, whereas a central patch and atrophy indicate advanced disease with moderate to severe loss of vision [31]. Previous studies reported that a decrease in VA was associated with a ring constriction smaller than 3° in radius [31,38], which closely approaches our microperimetric parafoveal inner ring area. From an anatomical perspective, when transitioning from the parafovea to the fovea region, rods decrease from being the predominant type of receptor cells, while cone cell bodies increase from a single layer to a tightly packed multilayer [39]. These findings may be explained by the deterioration of visual function once the retinal structure destruction touches the parafoveal ring, as this is the most central area that rods are predominant [40,41,42]. 

MP-3 is not without its restrictions. MP-3 testing is dependent on the subject’s physical and mental ability to cooperate, which may be challenging in patients with unstable fixation, in young children, and in those with insufficient clear optical media to allow the system to accurately track the fundus position relative to the stimuli being presented [43]. We have specifically excluded patients with the above limitations in our study. Moreover, we did not discern details of RP patients’ ONH because of the small number of patients in each group, and how RNFL thickness and ONH pallor influence VA still remains undetermined [11,12]. Further limitations of this research include the cross-sectional nature of the study. A longer observational period may be helpful in examining this indicator in IRD patients. Furthermore, because our study participants only consisted of Asian ethnicities and the patient number was relatively limited, further validation of our parameters in other ethnic groups with larger participant numbers may be required.

## 5. Conclusions

Identifying precise microperimetry parameters may provide complementary visual function measures in assessing central visual impairment severity in RP. Based on our findings, we established a microperimetric functional index according to the number of deep scotoma points at the parafoveal inner ring, which may represent a useful method to detect visual loss in RP patients. Four or more deep scotoma points at the parafoveal inner ring may be indicative of functional low vision. This study demonstrates the importance of microperimetry as an inevitable visual function assessment and may serve as a promising tool to monitor disease progression in future IRD studies.

## Figures and Tables

**Table 1:** Clinical characteristics of retinitis pigmentosa patients with fair and poor visual acuity.

	Fair VA (*n* = 21)	Poor VA (*n* = 18)	*p* Value
Age	44.57 ± 16.13	52.06 ± 14.16	0.135
Male gender, no. (%)	10 (47.6)	7 (38.9)	0.584
Optical coherence tomography			
Central macular thickness (μm)	240.19 ± 46.09	190.28 ± 48.95	0.002
Choroidal thickness (μm)	286.60 ± 83.65	256.06 ± 111.23	0.624
Loss of EZ integrity, no. (%)	5 (23.8)	14 (77.8)	0.001
Fundus autofluorescence pattern, no. (%)			0.024
Large ring	11 (52.4)	3 (16.7)	
Small ring	7 (33.3)	6 (33.3)	
Patchy atrophy	3 (14.3)	9 (50)	
Microperimetry			
Mean retinal sensitivity (dB)	14.98 ± 7.75	6.45 ± 7.24	0.001
Fixation points within 2° diameter circle (%)	68.29 ± 26.88	36.72 ± 31.96	0.002
Fixation points within 4° diameter circle (%)	87.43 ± 15.49	65.00 ± 27.45	0.005

**Table 2:** Location-specific microperimetric variables.

	Fair VA (*n* = 21)	Poor VA (*n* = 18)	*p* Value
Foveal center			
Deep scotoma points (no.)	0.62 ± 1.24	2.78 ± 2.32	0.002
Relative scotoma points (no.)	1.24 ± 1.51	1.06 ± 1.43	0.703
Normal points (no.)	3.14 ± 1.93	0.89 ± 1.49	<0.001
Inner ring			
Deep scotoma points (no.)	1.67 ± 2.35	5.22 ± 2.92	<0.001
Relative scotoma points (no.)	2.10 ± 2.26	1.44 ± 1.89	0.340
Normal points (no.)	4.24 ± 3.27	0.89 ± 1.75	<0.001
Outer ring			
Deep scotoma points (no.)	9.71 ± 7.04	15.50 ± 5.95	0.009
Relative scotoma points (no.)	5.14 ± 5.56	2.94 ± 4.90	0.202
Normal points (no.)	5.14 ± 6.51	0.72 ± 1.18	0.006

**Table 3:** Univariate and multivariate linear regression analysis of microperimetry parameters with logMAR VA.

	Univariate Analysis		Multivariate Linear Regression	
	Standardized β	*p* Value	Standardized β	*p* Value
Mean retinal sensitivity (dB)	−0.602	<0.001	−0.406	0.01
Fixation points within 2° diameter circle (%)	−0.534	<0.001		
Fixation points within 4° diameter circle (%)	−0.578	<0.001		
Deep scotoma points at fovea (no.)	0.591	<0.001		
Deep scotoma points at inner ring (no.)	0.580	<0.001	0.354	0.023
Deep scotoma points at outer ring (no.)	0.314	0.051		

## Data Availability

Data supporting this study are included within the article and Supplementary Materials.

## Supplementary Materials

The following supporting information can be downloaded at: https://www.mdpi.com/article/10.3390/diagnostics14232691/s1, Figure S1: Types and frequencies of gene mutations identified within our cohort.
