# Supplementary figures and images for: Parafoveal Microperimetric Retinal Sensitivity as a Key Parameter Associated with Vision Loss in Retinitis Pigmentosa
Source: Diagnostics (Basel). 2024 Nov 29;14(23):2691. doi: 10.3390/diagnostics14232691 (PMC11639803; doi:10.3390/diagnostics14232691)

**Supplementary Figure S1.** Types and frequencies of gene mutations identified within our cohort.

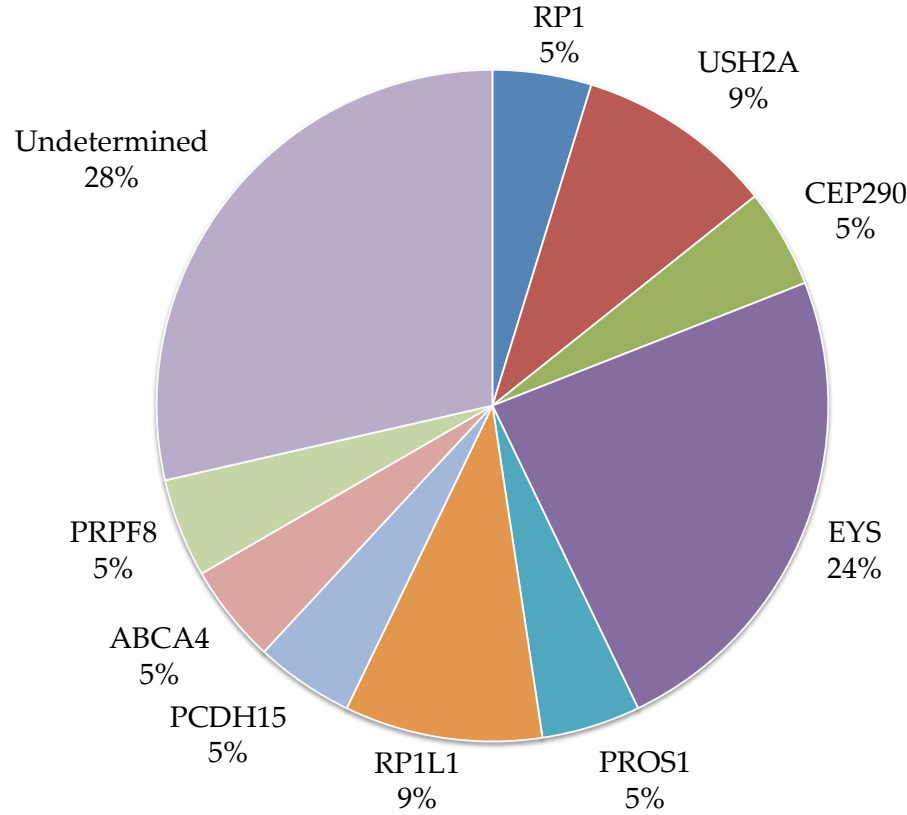

Supplement: Supplementary file 1 [file diagnostics-14-02691-s001.zip › Supplementary Figure S1.pdf]
